# Laparoscopy combined with transvaginal surgery for Herlyn–Werner–Wunderlich syndrome: A case report

**DOI:** 10.1097/MD.0000000000032264

**Published:** 2022-12-09

**Authors:** Gaoli Niu, Yanhong Zhai, Luhong Meng, Lingli Zhao, Nannan Liu, Xuejiao Xing, Xin Wen, Jingjing Chen

**Affiliations:** a Department of Gynecologic Oncology, The First Affiliated Hospital of Henan Polytechnic University (The Second People’s Hospital of Jiaozuo), Jiaozuo, China.

**Keywords:** Herlyn, laparoscopy, vaginal septum resection, Werner, Wunderlich syndrome

## Abstract

Herlyn–Werner–Wunderlich (HWW) syndrome is a rare congenital Mullerian duct anomaly disease that is characterized by a triad of symptoms, didelphys uterus, blind hemivagina, and ipsilateral renal agenesis. Herein, we reported a case from China.

**Case presentation:**

An 11-year-old patient presented to our hospital with lower abdominal pain and frequent urination. Computed tomography and magnetic resonance imaging revealed hematocolpos, uterine hemorrhage, didelphys uterus, and renal agenesis on the right side. Thus, the patient was diagnosed with HWW syndrome. Laparoscopic combined with transvaginal surgery to remove the vaginal septum, the symptoms of the lesion disappeared after the blood was discharged.

**Conclusion:**

Abnormal urination and other symptoms should be carefully examined in adolescent girls with abdominal pain not menarche, since they may be related to reproductive organ development disorders and other diseases. We recommend laparoscopy combined with transvaginal surgery to remove the oblique septum in HWW syndrome, which is rarely reported.

## 1. Introduction

Herlyn–Werner–Wunderlich (HWW) syndrome is a rare congenital Mullerian duct anomaly disease that is characterized by a triad of symptoms, didelphys uterus, blind hemivagina, and ipsilateral renal agenesis. HWW syndrome was first reported by Herlyn and Werner in 1971.^[1]^ Between January 1986 and March 2013, a total of 2238 cases of female reproductive malformations were reported, of which 79 cases were diagnosed with HWW syndrome. Hence, the incidence of this disease is about 0.1% to 3.5%.^[2]^ While the exact etiology of HWW syndrome is unknown, it may be related to the etiology of vice nephridium dysplasia during embryonic period, wherein the deputy nephridium does not reach the urogenital sinus causing a blind side.^[3]^ The clinical manifestations mainly include periodic abdominal pain, irregular vaginal bleeding or drainage, painful menstruation, and pelvic mass.^[4]^ Magnetic resonance imaging (MRI) and ultrasound examination play an important role in the diagnosis of HWW syndrome.^[4,5]^ Herein, we described the case of an 11-year-old girl who presented to our hospital with frequent urination and abdominal pain. The vaginal oblique septum was removed by laparoscopy combined with transvaginal surgery. Laparoscopy was performed under direct vision to detect double uterine malformations and thoroughly evaluate the pelvic cavity for adhesions or ectopic lesions.

## 2. Case presentation

This work has been carried out in accordance with the Declaration of Helsinki (2000) of the World Medical Association. This study was approved by the Ethic Committee of the First Affiliated Hospital of Henan Polytechnic University (the Second People’s Hospital of Jiaozuo) (Approval Number: KY2021-07-001), and written informed consent was obtained from the patient’s legal guardian/next of kin.

**Figure 1.**
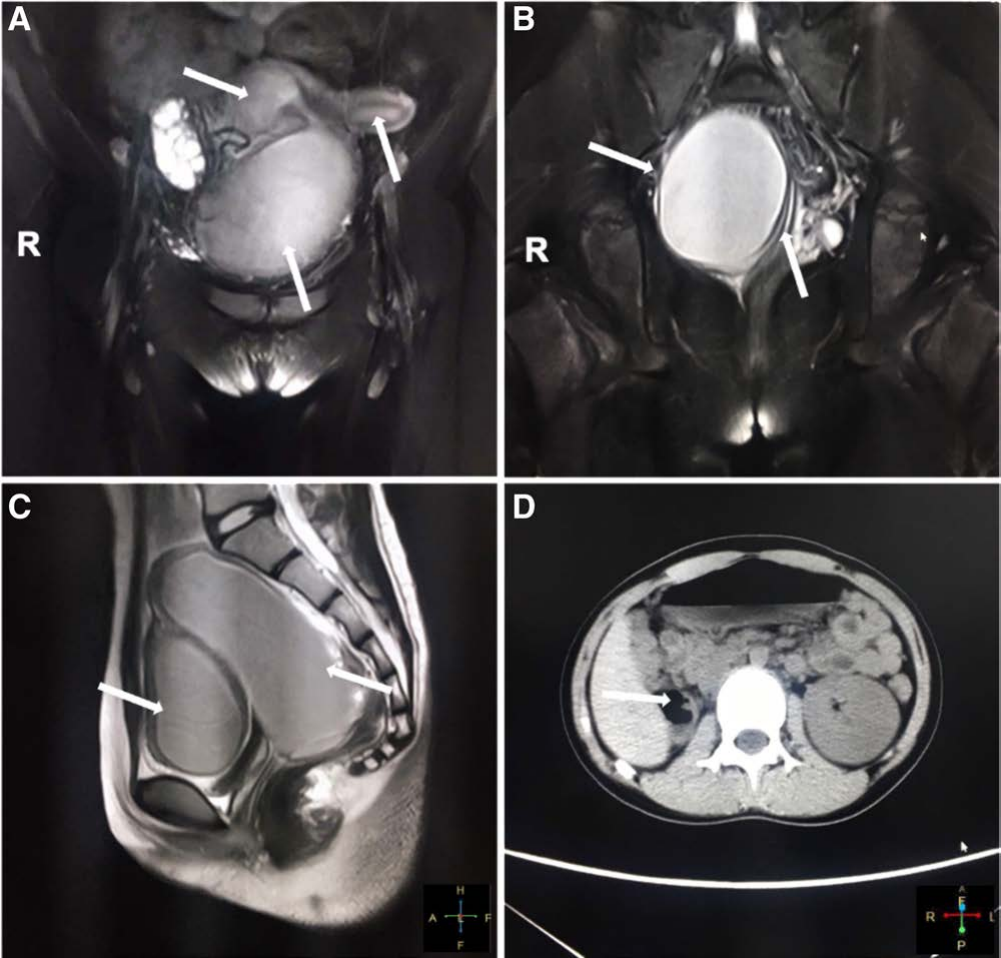
Magnetic resonance imaging and electron tomography findings of the patient. (A) Right uterus with increased hematoceles (upper right arrow), left uterus (upper left arrow), hematocolpos (lower arrow). (B) hematocolpos (upper right arrow). The left cervix was compressed into a narrow change due to hematocolpos (left arrow). (C) Right uterus with increased hematoceles (right arrow), hematocolpos (left arrow). (D) Non-visualization of the right kidney (right arrow).

**Figure 2.**
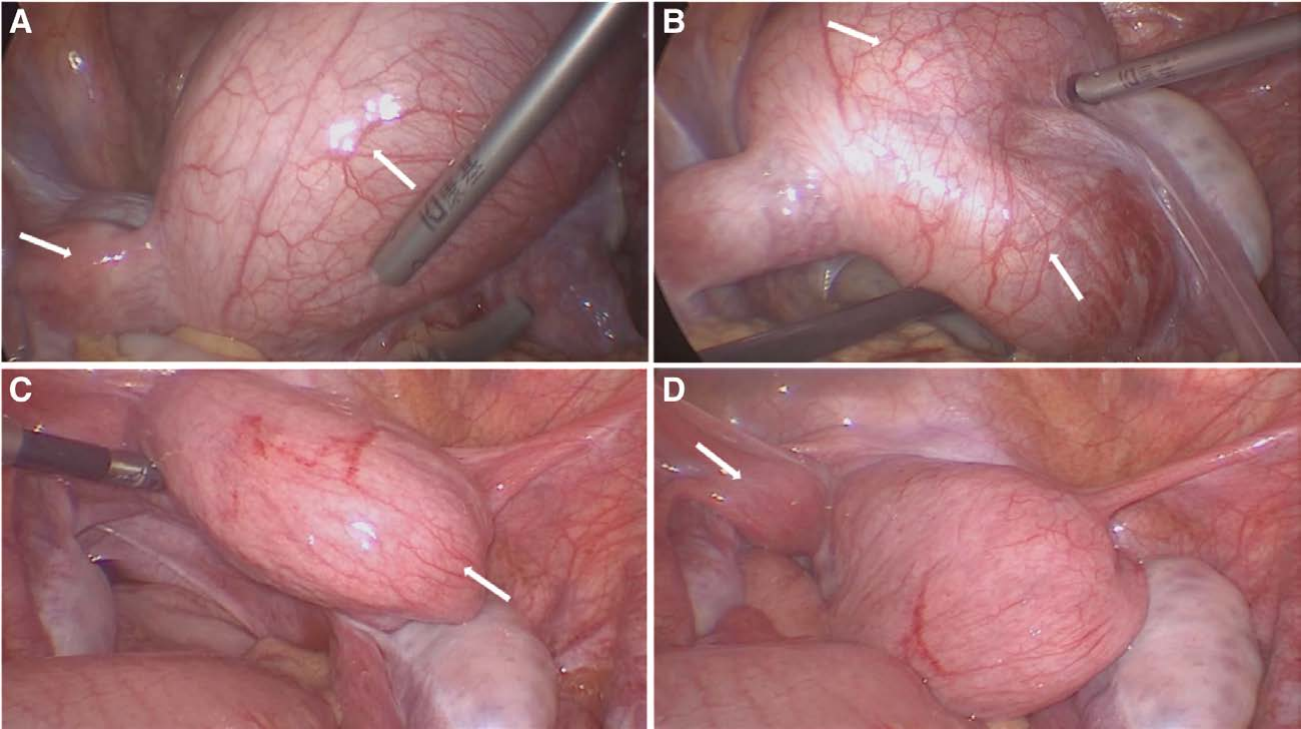
The uterus and adnexa were examined under laparoscope. (A) The arrow points to the biangular uterus. The smaller uterus is seen on the left and the enlarged uterus on the right. (B) The upper arrow indicates the enlarged uterus on the right, and the lower arrow indicates the vagina. (C) The arrow indicates the uterus after the hemorrhage was discharged. (D) The arrow indicates the left uterus.

**Figure 3.**
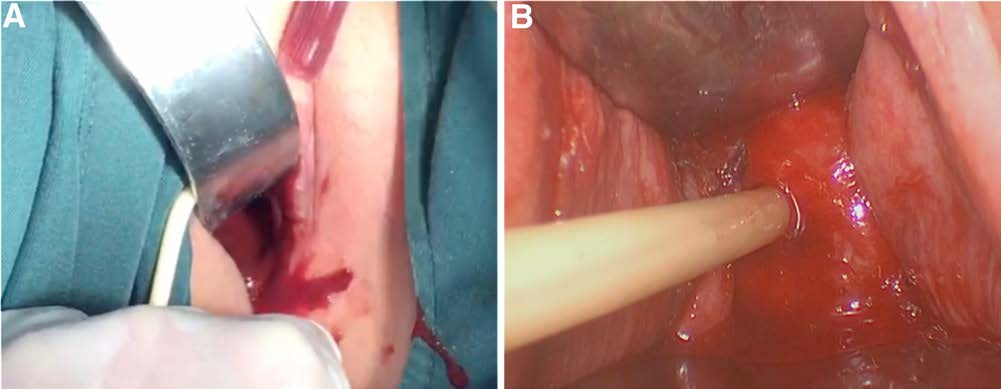
The uterus and adnexa were examined under laparoscope. (A) Hematocele was discharged after vaginal septum resection. B: Intrauterine bladder catheterization.

An 11-year-old girl was admitted to the First Affiliated Hospital of Henan Polytechnic University with complaints of intermittent lower abdominal pain and frequent urination for 6 months, which had worsened in the past 2 months. When the pain was severe, the patient also suffered from nausea, vomiting and other symptoms, but no fever. The day before the patient was admitted to our hospital, she underwent a color ultrasound at another hospital, which showed pelvic cysts and didelphys uterine malformations. Her right kidney was missing during maternal ultrasound at 8 months of gestation, but was not further examined after birth. She had normal postural development, with 156 centimeters height and 45 kilograms weight, and her secondary sexual characteristics such as breast development were normal. On the day of hospitalization, her vital signs were stable and anal examination revealed pelvic masses with a palpable bulge in the vagina. Her routine blood tests showed a slight increase in white blood cells at 10.95´10^9^/L and a differential count of 75.5% neutrophils. The CA19-9 level was 65.9 U/mL (normal range: 0–27 U/mL). The CA-125 level was 47.9 U/mL (normal range: 0–35 U/mL). The human epididymis protein 4 level was 93.6 pmol/L (normal range: 0–68 pmol/L). Pelvic MRI showed bicornuate uterine changes, with massive hemoperiosis in the right uterine cavity and hematocolpos, and endometrial lines in the left uterine cavity. The left cervix was compressed into a narrow change due to hematocolpos (Fig. 1A–C). Computed tomography (CT) of the abdomen revealed absence of right kidney (Fig. 1D). A diagnosis of HWW syndrome was made based on the patient’s history along with the findings of CT and pelvic MRI examinations. The patient underwent vaginal septum resection with vaginal hysteroscopy and laparoscopy. The position of the vaginal septum and the condition of the vagina were observed by hysteroscopy. A right oblique septum was identified. Laparoscopy showed that the uterus was didelphys and there was no communication between the two uterine cavities. The uterus on the right side was significantly enlarged to about 10 centimeters, with a noticeably enlarged vagina below. The size of the left uterus was significantly smaller than that of the normal uterus. No abnormalities were found in bilateral ovaries and fallopian tubes (Fig. 2). The septum was excised through the vagina, and about 500 milliliter (mL) of hemocele was discharged. After the operation, a right intrauterine balloon catheter was placed to drain the effusion. The catheter was removed 3 days later, and a total of 30 mL of light red fluid was drained (Fig. 3). No intraoperative or postoperative complications occurred, and the patient was discharged on the fourth day after surgery. The patient received regular follow-up after discharge, and no vaginal adhesions were found on vaginal examination 10 days after surgery. She had menstruation 1 month after surgery, with no abdominal pain or other complaints. The patient and her mother were very satisfied with our treatment.

## 3. Discussion

HWW syndrome consists of uterus didelphys, hemivaginal septum, and unilateral renal agenesis.^[1]^ The paramesonephric ducts and mesonephric ducts share a common origin and are closely related to each other. The development of paramesonephric ducts depends on the development of mesonephric ducts, which causes the dysplastic mesonephric ducts accompanied by vice in paramesonephric ducts. Therefore, the majority of patients with HWW syndrome also have ipsilateral renal agenesis.^[6]^ The clinical characteristics of HWW syndrome are mainly related to the location of the vaginal septum, whether the septum is perforated and the development of double uterus, as well as the fertility requirements of the patients. The majority of patients develop symptoms after menstruation in adolescence, and some cases are reported in prepubertal or neonatal period. In the present case, the patient was 11 years old and the onset was during adolescence. Dysmenorrhea or periodic abdominal pain are the main clinical symptoms of HWW syndrome.^[7]^ Different symptoms are associated with different classifications. Lan Zhu et al^[4]^ summarized the clinical characteristics of 79 patients and classified them into 2 different categories based on complete or incomplete obstruction of the hemivagina (Table 1).

**Table 1:** Classification of Herlyn–Werner–Wunderlich syndrome.

Class 1		Class 2	
Completely obstructed hemivagina		Incompletely obstructed hemivagina	
Subclass 1.1	Subclass 1.2	Subclass 2.1	Subclass 2.2
Blind hemivagina	Cervicovaginal atresia without atresia without uteri	Partial reabsorption of the vaginal septum	Incompletely obstructed hemivagina with communicating uteri

The present case belonged to subclass 1.1, and was characterized by double uterus and completely obstructed hemivagina. Therefore, the onset age was earlier. Pelvic ultrasound, CT and MRI are the main diagnostic methods for HWW syndrome. These examinations can clearly display the types of abnormal uterine and vaginal development, and show the obstructed hemivagina, vaginal hematoceles and ipsilateral uterine hematoceles, which is helpful for the classification of diseases and the judgment of pelvic conditions.^[5,8,9]^ Resection is the only effective treatment for HWW syndrome. The aim of the operation is to remove the obstruction of the reproductive tract and preserve the patient’s reproductive function. Hysteroscopy combined with laparoscopic surgery is advocated for the surgical treatment of patients with HWW syndrome, which facilitates clear diagnosis and screening of complicated pelvic endometriosis, and other pelvic and abdominal abnormalities.^[10]^ In the present case, hysteroscopy was used to enter the vagina to clearly observe the vaginal oblique septum, followed by vaginal oblique septum resection under the supervision of laparoscopy to observe the development of the vagina, uterus and ovary in the abdominal cavity. No intraperitoneal adhesions or pelvic endometriosis were observed. The patient recovered well after surgery. Postoperative natural pregnancy rate of HWW syndrome patients is related to the presence of secondary pelvic infection, endometriosis and pelvic adhesion before surgery. Pregnancy is possible in bilateral uterus after surgical treatment of HWW syndrome, and the postoperative pregnancy rate of infertile patients is 87%, among which 62% of the patients can deliver at term, but 23% of the patients suffer from the risk of abortion and 15% of the patients could have premature delivery.^[11,12]^ Therefore, our patient needs long-term follow-up to observe any future pregnancy.

HWW syndrome is a rare disease. Early diagnosis is of great significance for preserving the fertility of patients, and surgery is the only effective treatment. Therefore, we recommend laparoscopic vaginal septum resection combined with transvaginal hysteroscopy for the treatment of HWW syndrome.

## Author contributions

Gaoli Niu and Yanhong Zhai carried out the studies, participated in collecting data, and drafted the manuscript. Luhong Meng and Lingli Zhao and Nannan Liu performed the statistical analysis and participated in its design. Xuejiao Xing and Xin Wen and Jingjing Chen participated in acquisition, analysis, or interpretation of data and draft the manuscript. All authors read and approved the final manuscript.

**Conceptualization:** Gaoli Niu, Yanhong Zhai, Luhong Meng, Lingli Zhao, Nannan Liu.

**Formal analysis:** Luhong Meng, Lingli Zhao, Nannan Liu, Xuejiao Xing, Xin Wen, Jingjing Chen.

**Funding acquisition:** Yanhong Zhai.

**Investigation:** Gaoli Niu, Yanhong Zhai, Xuejiao Xing, Xin Wen, Jingjing Chen.

**Methodology:** Gaoli Niu, Yanhong Zhai, Xuejiao Xing, Xin Wen, Jingjing Chen.

**Writing – original draft:** Gaoli Niu, Yanhong Zhai.

**Writing – review & editing:** Gaoli Niu, Yanhong Zhai, Luhong Meng, Lingli Zhao, Nannan Liu, Xuejiao Xing, Xin Wen, Jingjing Chen.
